# Integrative Single-Cell and Radiomics Analyses Identify N-Acetylneuraminate Synthase (*NANS*)-High Signature Associated with Adverse Prognosis and Immune Remodeling in Glioblastoma

**DOI:** 10.3390/ijms27219635

**Published:** 2026-10-28

**Authors:** Jianan Chen, Qiong Wu, Hannah M. Cardenas, Kaitlyn N. Bernard, Anders E. Berglund, Yu Sun, Robert J. Macaulay, James J. Mulé, Arnold B. Etame

**Affiliations:** 1 Department of Neuro-Oncology, H. Lee Moffitt Cancer Center and Research Institute, Tampa, FL 33612, USA; 2 Department of Quantitative Health Sciences, Division of Computational Biology, Mayo Clinic 4500 San Pablo Road South, Jacksonville, FL 32224, USA; 3 Departments of Anatomic Pathology, H. Lee Moffitt Cancer Center and Research Institute, 12902 Magnolia Drive, Tampa, FL 33612, USA; 4 Department of Immunology, H. Lee Moffitt Cancer Center and Research Institute, 12902 Magnolia Drive, Tampa, FL 33612, USA

**Keywords:** glioblastoma, *NANS*, radiomics, tumor-associated macrophages, tumor microenvironment, LGALS9

## Abstract

Glioblastoma (GBM) is characterized by marked heterogeneity and an immunosuppressive microenvironment. N-acetylneuraminate synthase (*NANS*) contributes to tumor-associated metabolic regulation, but its relationship with GBM and its immune profile remains unclear. This study aimed to characterize the prognostic and immune relevance of *NANS* and develop an MRI radiomics model for noninvasive prediction of *NANS*-high GBM. Prognostic genes were screened across TCGA-GBM and two CGGA cohorts. Bulk transcriptomic and single-cell RNA sequencing analyses were used to evaluate *NANS* expression, patient survival association, and cellular localization. *NANS*-high bone marrow-derived macrophages (BMDMs) were characterized using functional enrichment and CellChat analyses. A *NANS*-high BMDM signature was validated in TCGA-GBM for immune infiltration and ligand–receptor axis activity. Drug sensitivity and CMap analyses were performed. MRI radiomics features were extracted from preprocessed T1 contrast-enhanced and FLAIR images, and an elastic net logistic regression model was constructed to predict *NANS*-high BMDM signature status. *NANS* was identified as a prognostic macrophage-associated biomarker in GBM and was enriched in BMDMs. *NANS*-high BMDMs showed enrichment of mTORC1 signaling, oxidative phosphorylation, unfolded protein response, glycolysis, hypoxia, and protein secretion programs. Cell–cell communication analysis revealed enhanced outgoing signaling toward malignant tumor states and T-cell subsets, with LGALS9-centered interactions representing the dominant differential communication program. In TCGA-GBM, the *NANS*-high BMDM signature correlated with increased macrophage infiltration, broad immune infiltration, and elevated LGALS9 and PLAU axis scores. Drug sensitivity analyses suggested potential vulnerabilities involving PI3K/mTOR and MAPK/ERK pathways. The radiomics model selected six MRI features and distinguished *NANS*-high from *NANS*-low tumors with an AUC of 0.795 (95% CI, 0.652–0.939); bootstrap validation showed a mean AUC of 0.793 (95% CI, 0.637–0.922). *NANS*-high BMDMs define a prognostically adverse and potentially targetable macrophage-associated phenotype in GBM. MRI radiomics may noninvasively predict this *NANS*-high BMDM state, supporting imaging-based risk stratification and therapeutic selection.

## 1. Introduction

Glioblastoma (GBM) remains the most lethal primary malignancy of the adult central nervous system, with poor survival despite maximal safe resection, radiotherapy, and temozolomide-based chemotherapy [1,2]. Therapeutic resistance in GBM is largely driven by profound biological heterogeneity, involving genetic, epigenetic, developmental, and microenvironmental programs [3,4]. Recent single-cell transcriptomic studies have shown that malignant GBM cells exist in distinct and plastic cellular states, including neural progenitor-like, oligodendrocyte progenitor-like, astrocyte-like, and mesenchymal-like programs [3]. This cellular-state diversity provides an important framework for understanding tumor progression, treatment resistance, and tumor–microenvironment interactions.

The GBM immune microenvironment is characterized by limited effective lymphocyte infiltration and abundant immunosuppressive myeloid cells. Tumor-associated macrophages and microglia represent a major immune component of GBM and contribute to immune evasion, angiogenesis, tumor progression, and therapeutic resistance [5,6]. These macrophage-lineage populations include brain-resident microglia and recruited bone marrow-derived macrophages (BMDMs), which differ in ontogeny, transcriptional features, and functional roles within the tumor ecosystem [7]. Given the limited clinical benefit of immune checkpoint blockade in GBM, a more detailed understanding of macrophage heterogeneity and macrophage-mediated tumor–immune communication is needed to identify clinically relevant immune states and therapeutic vulnerabilities [8].

Beyond macrophage polarization, metabolic and glycosylation-related programs may further shape myeloid-cell function within the tumor microenvironment. Aberrant sialylation can modulate tumor–immune interactions through Siglec–sialic acid signaling, thereby contributing to immune evasion and suppression of anti-tumor responses [9]. N-acetylneuraminate synthase (*NANS*), a key enzyme involved in de novo sialic acid biosynthesis, may participate in tumor-associated sialylation programs; however, its role in the glioma ecosystem remains poorly defined [10]. Whether *NANS* marks a distinct macrophage-associated state, and whether this state is linked to malignant cellular programs, T-cell subsets, immune infiltration, and therapeutic vulnerability, has not been systematically investigated.

Single-cell RNA sequencing provides a powerful approach to resolve the cellular localization and functional context of candidate biomarkers within the GBM ecosystem. Prior single-cell studies have mapped malignant cell states, immune cell heterogeneity, and intercellular communication networks in human gliomas [5]. However, transcriptomic profiling is tissue-dependent and may not be available before treatment. MRI is routinely used in GBM diagnosis and management, and radiomics enables high-throughput extraction of quantitative imaging features that may reflect underlying tumor heterogeneity. Because macrophage states are influenced by spatially heterogeneous tumor niches, including edema, necrosis, and vascular remodeling, radiomics may provide a noninvasive surrogate for selected macrophage-associated molecular phenotypes [11,12].

In this study, we integrated multi-omics and radiomics to systematically dissect the role of *NANS* in the GBM TME. We first delineated its cellular localization and intercellular communication networks at single-cell resolution, then established its clinical relevance and therapeutic implications in bulk cohorts and finally constructed a radiomics-based imaging biomarker to enable noninvasive patient stratification for a *NANS*-associated specific immune state.

## 2. Results

### 2.1. NANS Is Associated with Poor Prognosis and Is Highly Expressed in BMDMs

**Figure 1:**
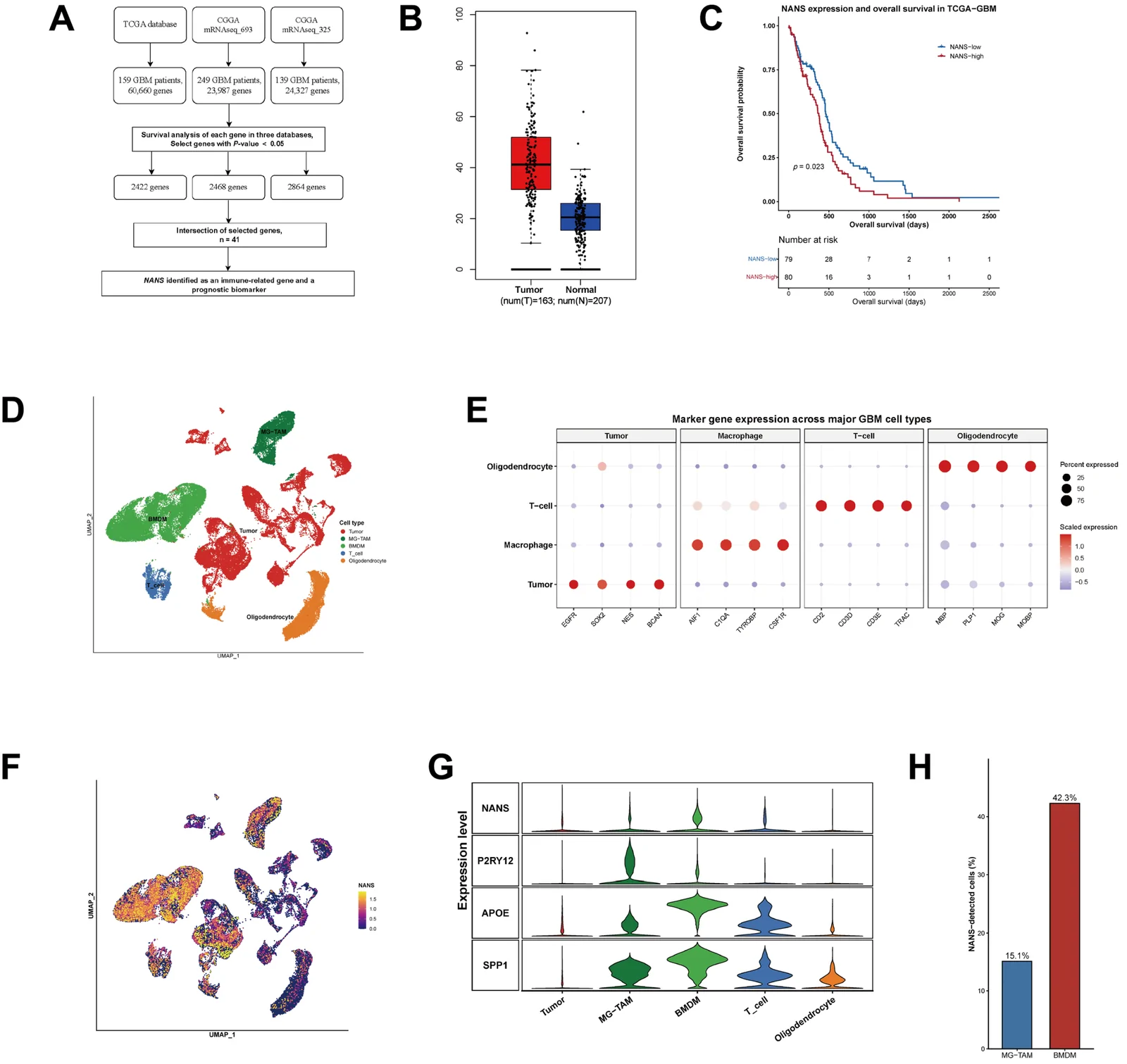
Identification of *NANS* as a prognostic and macrophage-associated biomarker in glioblastoma. (**A**) Workflow for prognostic gene screening across TCGA-GBM, CGGA mRNAseq_693, and CGGA mRNAseq_325, identifying 41 overlapping genes. (**B**) *NANS* expression in GBM and normal brain tissues using GEPIA. (**C**) Kaplan–Meier analysis of overall survival according to *NANS* expression in TCGA-GBM. (**D**) UMAP of major GBM cell populations, including tumor cells, MG-TAMs, BMDMs, T cells, and oligodendrocytes. (**E**) Dot plot of canonical marker genes across major cell types. (**F**) Feature plot of *NANS* expression in the single-cell dataset. (**G**) Violin plots showing *NANS* and representative macrophage-associated genes, including *P2RY12*, *APOE*, and *SPP1*, across major cell types. (**H**) Bar plot showing the proportion of *NANS*-detected cells in macrophage subsets. *NANS*-detected cells accounted for 15.1% of MG-TAMs and 42.3% of BMDMs.

To identify prognostically relevant genes in GBM, survival analyses were performed across TCGA-GBM, CGGA mRNAseq_693, and CGGA mRNAseq_325 cohorts. A total of 2422, 2468, and 2864 survival-associated genes were identified in the three cohorts, respectively, and 41 overlapping prognostic genes were retained. Among these 41 genes (Supplementary Table S1), *NANS* was selected for further hypothesis-driven investigation based on its role in sialic acid biosynthesis and its potential relevance to the tumor immune microenvironment, rather than statistical superiority over the other candidates (Figure 1A). *NANS* expression was higher in GBM tissues than in normal brain tissues (Figure 1B), and high *NANS* expression was associated with significantly worse overall survival in TCGA-GBM patients (*p* = 0.023; Figure 1C). Stratified analyses were further performed according to IDH status and MGMT-promoter methylation status. NANS expression did not differ significantly between IDH-wildtype and IDH-mutant tumors or between MGMT-methylated and unmethylated tumors (Supplementary Figure S1A,B). High NANS expression remained associated with poorer overall survival in IDH-wildtype GBM (HR = 1.53, 95% CI: 1.01–2.30; *p* = 0.042) and in the MGMT-methylated subgroup (HR = 3.01, 95% CI: 1.43–6.36; *p* = 0.0024), whereas no significant association was observed in the IDH-mutant or MGMT-unmethylated subgroups (Supplementary Figure S1C–F).

Single-cell analysis identified five major cellular populations, including tumor cells, MG-TAMs, BMDMs, T cells, and oligodendrocytes, with cell annotations supported by canonical marker genes (Figure 1D,E). *NANS* expression was mainly localized to the macrophage compartment and was particularly enriched in BMDMs (Figure 1F,G). Within macrophage-lineage cells, the proportion of *NANS*-detected cells was higher in BMDMs than in MG-TAMs (42.3% vs. 15.1%, *p* < 0.001; Figure 1H).

### 2.2. NANS-High BMDMs Are Characterized by Metabolic Activation and Protein-Processing Programs

**Figure 2:**
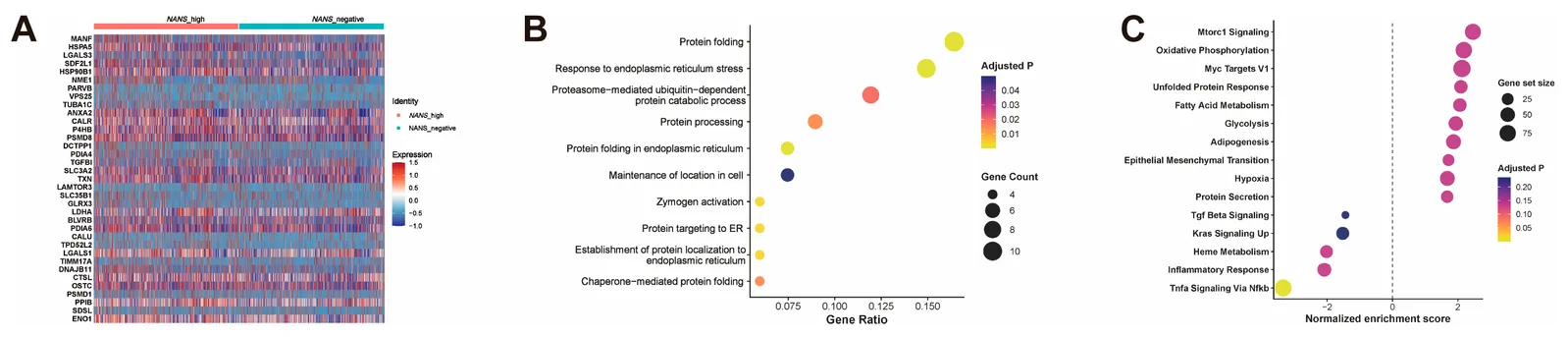
Functional characterization of *NANS*-high BMDMs in glioblastoma. (**A**) Heatmap of representative differentially expressed genes between *NANS*-high and *NANS*-low BMDMs. (**B**) Gene Ontology enrichment analysis of genes upregulated in *NANS*-high BMDMs. (**C**) Hallmark gene set enrichment analysis comparing *NANS*-high and *NANS*-low BMDMs. Positive normalized enrichment scores indicate pathways enriched in *NANS*-high BMDMs, whereas negative scores indicate pathways enriched in *NANS*-low BMDMs. Dot size represents gene set size, and color indicates adjusted *p* value.

To define the biological features of *NANS*-high macrophages, we compared the transcriptional profiles of *NANS*-high and *NANS*-low BMDMs. Heatmap analysis showed that *NANS*-high BMDMs exhibited a distinct expression pattern, with increased expression of genes related to protein folding, endoplasmic reticulum stress, secretion, and cellular stress adaptation, including *MANF*, *HSPA5*, *LGALS3*, *HSP90B1*, *CALR, PDIA4*, and *LGALS1* (Figure 2A).

Functional enrichment analysis further showed that genes enriched in *NANS*-high BMDMs were mainly associated with protein folding, response to endoplasmic reticulum stress, proteasome-mediated protein catabolism, protein processing, and protein targeting to the endoplasmic reticulum (Figure 2B). GSEA revealed positive enrichment of MTORC1 signaling, oxidative phosphorylation, MYC targets, unfolded protein response, fatty acid metabolism, glycolysis, adipogenesis, epithelial–mesenchymal transition, hypoxia, and protein secretion in *NANS*-high BMDMs (Figure 2C). In contrast, inflammatory response, TNFα signaling via NF-κB, heme metabolism, KRAS signaling, and TGF-β signaling were negatively enriched.

### 2.3. NANS-High BMDMs Show Increased CellChat-Inferred Communication with Tumor and T-Cell Populations

**Figure 3:**
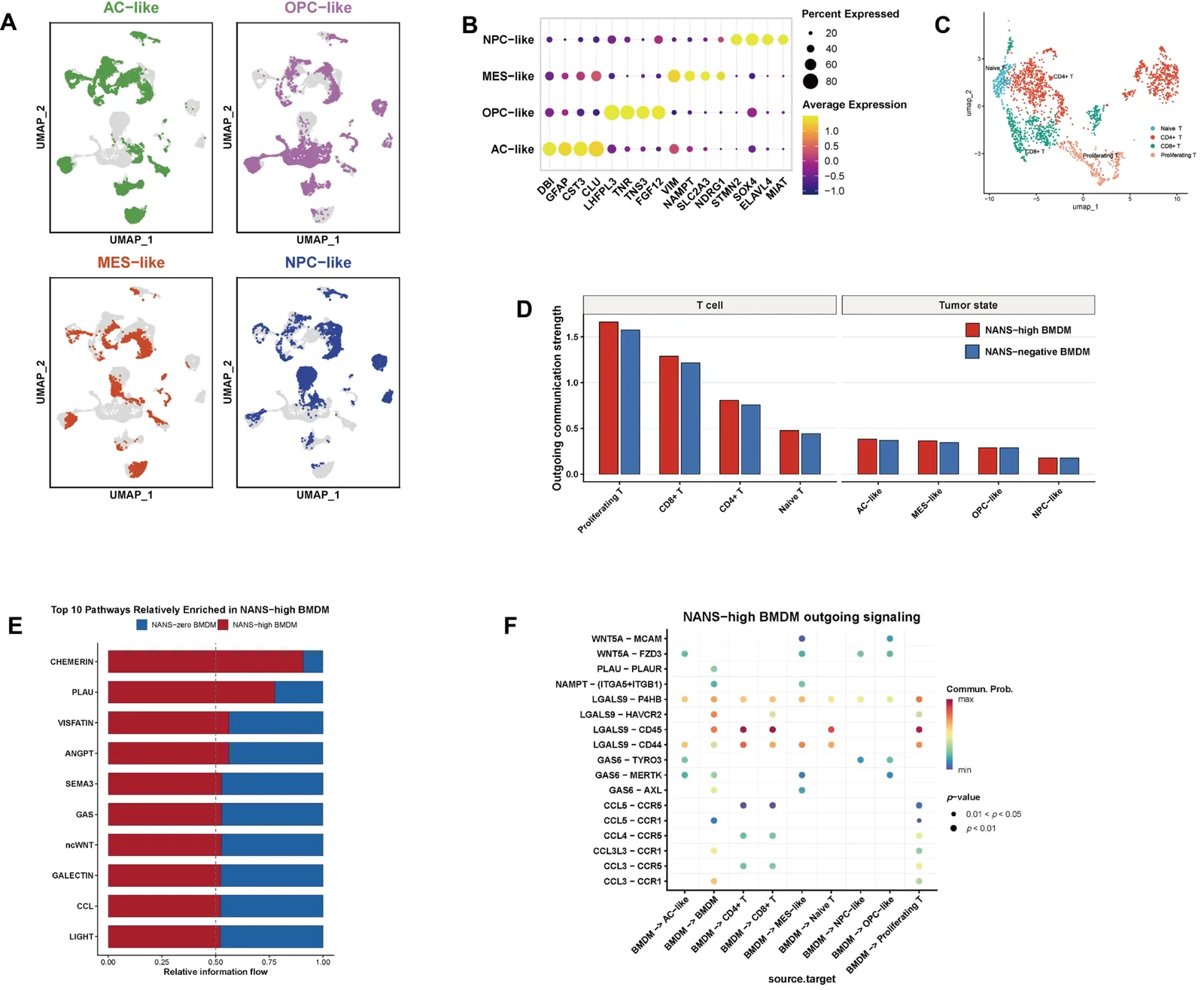
CellChat-inferred intercellular communication associated with *NANS*-high BMDMs in glioblastoma. (**A**) UMAP of malignant tumor cells classified into AC-like, OPC-like, MES-like, and NPC-like states. (**B**) Dot plot of marker genes used to annotate malignant tumor states. (**C**) UMAP of T-cell subpopulations, including naïve T cells, CD4+ T cells, CD8+ T cells, and proliferating T cells. (**D**) CellChat-inferred outgoing communication strength from *NANS*-high and *NANS*-low BMDMs to T-cell subsets and malignant tumor states. (**E**) Relative information flow of the top 10 signaling pathways with higher inferred activity in *NANS*-high BMDMs. (**F**) Bubble plot of ligand–receptor pairs showing the largest inferred differences between *NANS*-high and *NANS*-low BMDMs across interactions with malignant tumor states and T-cell subsets.

To further investigate the interaction between *NANS*-high BMDMs and other cellular compartments, malignant tumor cells were classified into AC-like, OPC-like, MES-like, and NPC-like states, while T cells were annotated as naïve T, CD4+ T, CD8+ T, and proliferating T subsets (Figure 3A–C). Compared with *NANS*-low BMDMs, *NANS*-high BMDMs were inferred by CellChat to have higher outgoing communication strength toward both T-cell subsets and malignant tumor states, particularly toward proliferating T cells, CD8+ T cells, and CD4+ T cells (Figure 3D). Pathway-level comparison further identified several signaling pathways preferentially enriched in *NANS*-high BMDMs, including CHEMERIN, PLAU, VISFATIN, and ANGPT signaling (Figure 3E). Ligand–receptor analysis further identified several differential outgoing signals from *NANS*-high BMDMs. Among these, LGALS9-centered interactions showed the largest inferred differences, including LGALS9–CD45, LGALS9–CD44, LGALS9–P4HB, and LGALS9–HAVCR2, with predicted interactions involving CD4+ T cells, CD8+ T cells, proliferating T cells, and BMDMs (Figure 3F).

### 2.4. Development and Characterization of a NANS-High BMDM Signature

**Figure 4:**
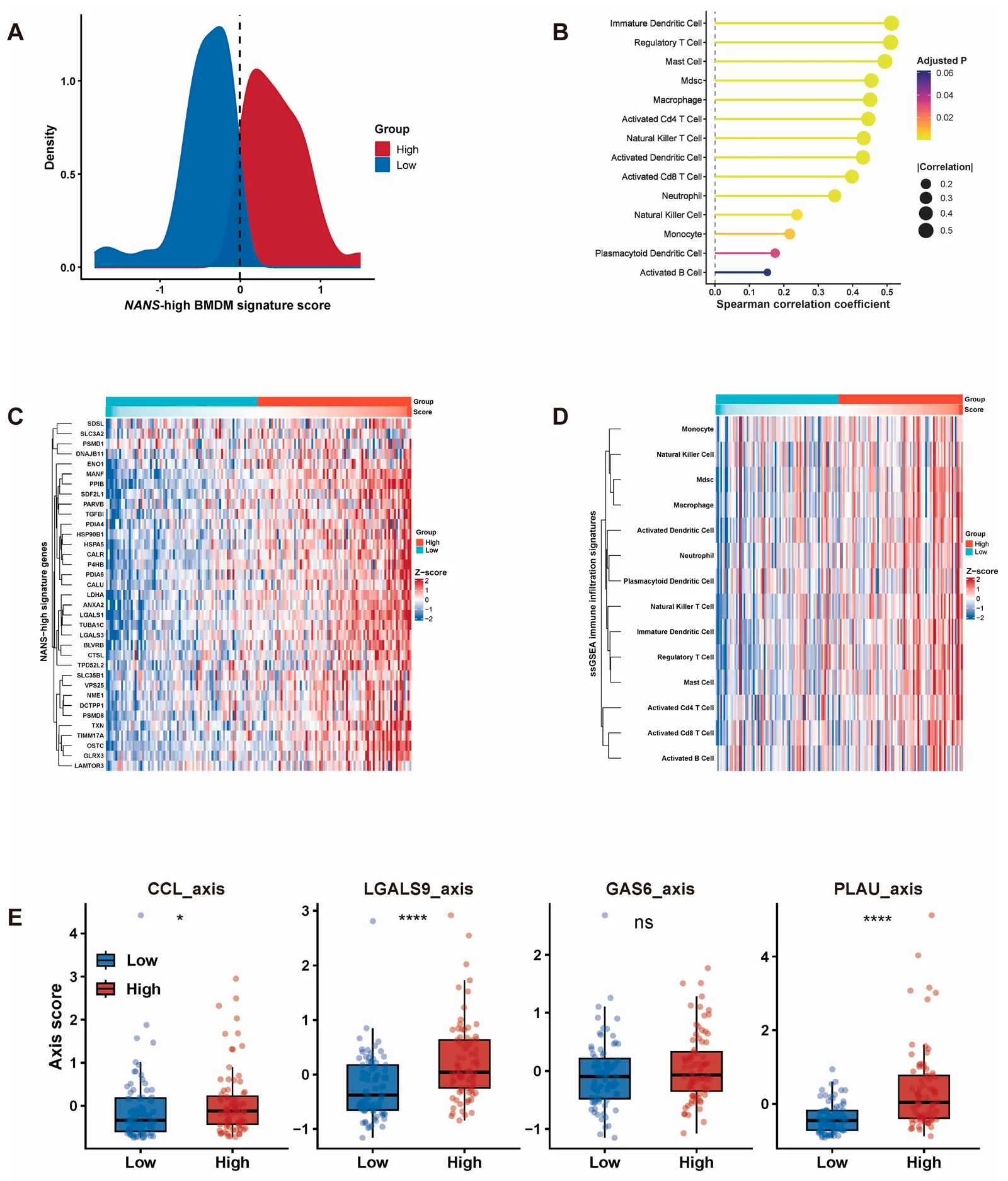
Bulk transcriptomic validation of the *NANS*-high BMDM signature and its association with immune infiltration in TCGA-GBM. (**A**) Distribution of the *NANS*-high BMDM signature score in TCGA-GBM patients and stratification into signature-high and signature-low groups. (**B**) Spearman correlations between the *NANS*-high BMDM signature score and ssGSEA-derived immune cell signatures. (**C**) Heatmap of *NANS*-high BMDM signature genes across TCGA-GBM patients. (**D**) Heatmap of ssGSEA-derived immune infiltration signatures stratified by signature score. (**E**) Comparison of selected ligand–receptor axis scores between signature-high and signature-low groups. Statistical significance: ns, not significant; * *p* < 0.05; **** *p* < 0.0001.

To translate the single-cell findings into bulk transcriptomic data, we constructed a *NANS*-high BMDM signature based on genes enriched in *NANS*-high BMDMs and calculated a signature score for each TCGA-GBM patient. Patients were then stratified into high- and low-score groups according to the signature score distribution (Figure 4A). The signature genes showed coordinated upregulation in the high-score group, supporting the consistency of this macrophage-associated transcriptional program in bulk GBM samples (Figure 4C).

ssGSEA-based immune infiltration analysis showed that the *NANS*-high BMDM signature score was positively correlated with multiple immune cell signatures, including immature dendritic cells, regulatory T cells, mast cells, MDSCs, macrophages, and activated CD4+ T cells (Figure 4B). To determine whether the *NANS*-high BMDM signature primarily reflected macrophage abundance, we compared the signature score with MCP-counter-derived monocytic lineage abundance. No significant correlation was observed (Spearman ρ = 0.074, *p* = 0.352), suggesting that the *NANS*-high BMDM signature captures a distinct macrophage transcriptional state rather than simply reflecting macrophage abundance. Consistently, the heatmap showed broadly increased immune cell signature scores in the high-score group, particularly involving myeloid cells, dendritic cells, regulatory T cells, and activated T-cell populations (Figure 4D). In addition, LGALS9 and PLAU axis scores were significantly increased in the high-score group, whereas the GAS6 axis showed no significant difference (Figure 4E). These findings support that the *NANS*-high BMDM signature captures key macrophage-associated communication programs in bulk GBM, especially LGALS9- and PLAU-centered signaling.

### 2.5. Drug Sensitivity Analysis Reveals Potential Therapeutic Vulnerabilities

**Figure 5:**
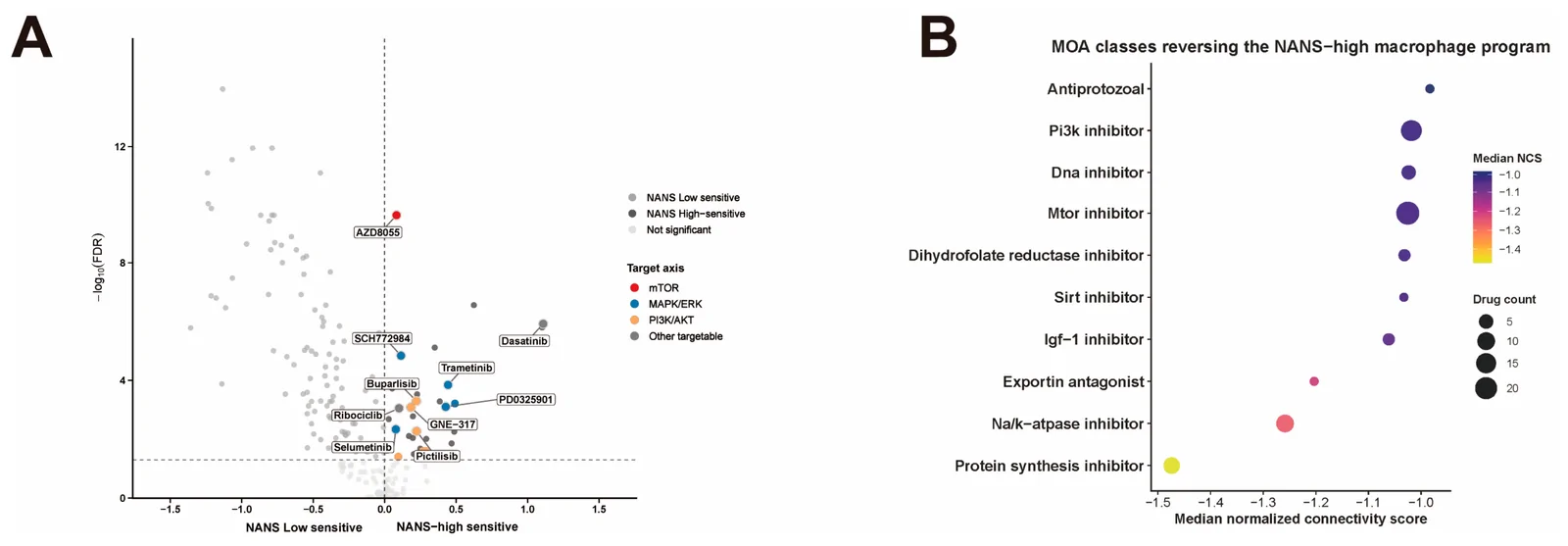
Drug sensitivity and CMap analysis reveal potential therapeutic vulnerabilities in *NANS*-high GBM patients. (**A**) Volcano plot of differential predicted drug sensitivity between *NANS*-high and *NANS*-low GBM groups. Selected compounds are labeled and grouped by major target pathways. (**B**) Mechanism-of-action enrichment analysis of compounds predicted by CMap to reverse the *NANS*-high macrophage-associated transcriptional program. Negative normalized connectivity scores indicate predicted reversal effects.

To explore potential therapeutic vulnerabilities associated with the *NANS*-high BMDM phenotype, drug sensitivity analysis was performed by comparing predicted drug responses between *NANS*-high and *NANS*-low groups. Several compounds showed preferential sensitivity in the *NANS*-high group, including AZD8055, SCH772984, Trametinib, PD0325901, GNE-317, Buparlisib, Pictilisib, Dasatinib, Ribociclib, and Selumetinib (Figure 5A). Connectivity analysis further identified drug mechanism-of-action classes with negative normalized connectivity scores, indicating potential reversal of the *NANS*-high macrophage program. The most enriched classes included protein synthesis inhibitors, Na+/K+-ATPase inhibitors, exportin antagonists, IGF-1 inhibitors, mTOR inhibitors, PI3K inhibitors, and DNA inhibitors (Figure 5B). These computational findings suggest that the *NANS*-high BMDM-associated phenotype may be associated with predicted therapeutic vulnerabilities involving mTOR/PI3K signaling, MAPK/ERK signaling, protein synthesis, and cellular stress regulation.

### 2.6. MRI Radiomics Enables Noninvasive Prediction of NANS-High BMDM Status

**Figure 6:**
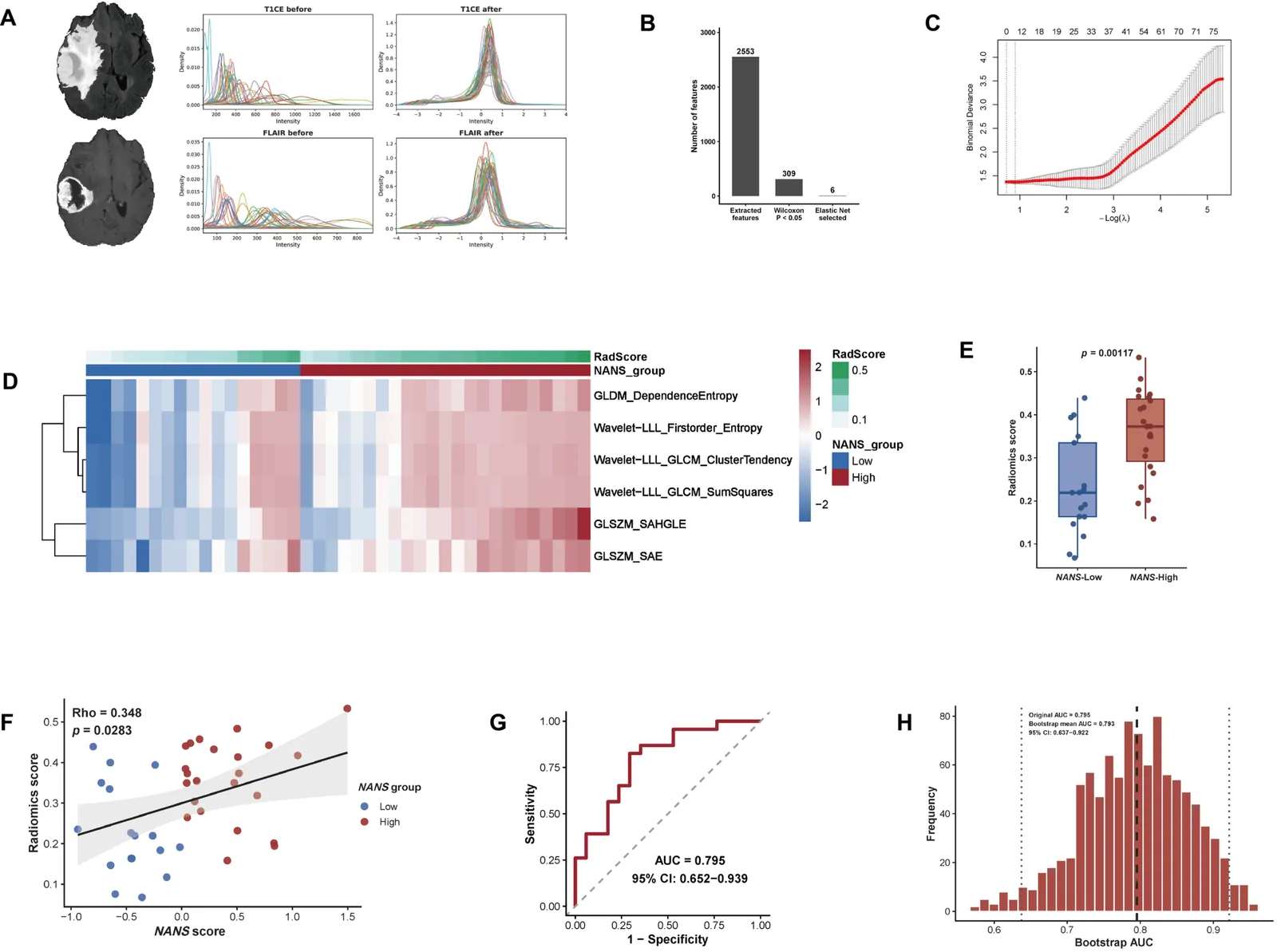
MRI radiomics model predicts *NANS*-high status in glioblastoma. (**A**) MRI preprocessing and intensity normalization workflow. (**B**) Radiomics feature reduction from 2553 initial features to 309 after Wilcoxon filtering and 6 after elastic net selection. (**C**) Cross-validation curve for elastic net logistic regression and selection of the optimal regularization parameter. (**D**) Heatmap of the 6 selected radiomics features across *NANS*-high and *NANS*-low patients. (**E**) Comparison of radiomics scores between *NANS*-high and *NANS*-low groups. (**F**) Spearman correlation between radiomics score and *NANS* score. (**G**) ROC curve of the radiomics model (AUC = 0.795, 95% CI: 0.652–0.939). (**H**) Bootstrap validation of model performance (mean AUC = 0.793, 95% CI: 0.637–0.922).

To investigate whether the *NANS*-high BMDM-associated molecular phenotype could be inferred noninvasively from routine MRI, we performed radiomics analysis using TCIA imaging data from 40 GBM patients with matched transcriptomic-derived *NANS*-BMDM signature scores. T1 contrast-enhanced (T1CE) and FLAIR images were first subjected to image preprocessing and intensity normalization. The normalized images were then used for radiomics feature extraction and downstream model construction (Figure 6A). A total of 2553 radiomics features were initially extracted from the MRI data. Feature filtering using the Wilcoxon test retained 309 candidate features associated with *NANS*-high versus *NANS*-low status. Elastic net modeling further reduced these candidates to six final radiomics features (Figure 6B,C). These selected features included GLDM_DependenceEntropy, Wavelet-LLL_Firstorder_Entropy, Wavelet-LLL_GLCM_ClusterTendency, Wavelet-LLL_GLCM_SumSquares, GLSZM_SAHGLE, and GLSZM_SAE. These features primarily capture image intensity heterogeneity, texture complexity, and gray-level spatial organization, suggesting that the *NANS*-high phenotype may be associated with increased intratumoral structural heterogeneity on MRI. Heatmap analysis showed that the selected radiomics features were generally elevated in the *NANS*-high group compared with the *NANS*-low group and were concordant with increasing RadScore values (Figure 6D). Consistently, the RadScore was significantly higher in *NANS*-high tumors than in *NANS*-low tumors (*p* = 0.00117; Figure 6E). In addition to the binary group comparison, RadScore was positively correlated with the continuous *NANS*-BMDM signature score across the 40 patients (Spearman rho = 0.348, *p* = 0.0283; Figure 6F). The radiomics model achieved an area under the receiver operating characteristic curve (AUC) of 0.795 for discriminating *NANS*-high from *NANS*-low tumors, with a 95% confidence interval of 0.652–0.939 (Figure 6G). Bootstrap resampling further supported the robustness of model performance, with a bootstrap mean AUC of 0.793 and a 95% confidence interval of 0.637–0.922 (Figure 6H).

## 3. Discussion

In this study, we integrated bulk transcriptomic analysis, single-cell RNA sequencing, drug sensitivity prediction, and MRI radiomics to characterize the biological and clinical significance of *NANS* in GBM. We identified *NANS* as a prognostically relevant gene preferentially enriched in BMDMs within the GBM microenvironment. Single-cell analysis further showed that *NANS*-high BMDMs represented a transcriptionally distinct macrophage state characterized by metabolic activation, protein-processing and stress-response programs, intensified communication with malignant tumor states and T-cell subsets, and LGALS9/PLAU-centered signaling. By translating this single-cell-defined state into bulk GBM transcriptomes, we found that the *NANS*-high BMDM signature was associated with broad immune infiltration and macrophage-related communication programs. The drug prediction and radiomics analyses suggested that this immune state may be linked to therapeutic vulnerabilities and may be inferred noninvasively from routine MRI. Together, these findings suggest *NANS*-high BMDMs as a macrophage-associated immune state with potential biological, therapeutic, and imaging relevance in GBM.

A central implication of our study is that *NANS* is not merely a bulk prognostic marker but is preferentially associated with a specific macrophage-lineage compartment in GBM. This distinction is biologically important because tumor-associated macrophages in GBM are not a uniform population; resident microglia and recruited BMDMs differ in ontogeny, transcriptional programs, spatial distribution, and functional contribution to the tumor ecosystem [7,13,14]. Prior single-cell studies have shown that specific myeloid-cell states, rather than total macrophage abundance alone, may carry distinct prognostic and therapeutic implications. In this context, the enrichment of *NANS* in BMDMs suggests that *NANS* may mark a recruited macrophage-associated state with potential relevance to the immunosuppressive GBM microenvironment. Therefore, focusing on *NANS*-high BMDMs allows a more refined interpretation of macrophage heterogeneity and avoids treating the broader TAM compartment as a single biological entity.

The functional profile of *NANS*-high BMDMs further supports the interpretation that this population represents an activated and stress-adapted macrophage state rather than a passive marker-defined subset. The enrichment of mTORC1 signaling, oxidative phosphorylation, unfolded protein response, glycolysis, hypoxia, and protein secretion suggests increased metabolic and proteostatic demands [15,16]. Such programs may be particularly relevant for macrophages engaged in active communication with tumor and immune compartments, because secretory and remodeling functions require substantial biosynthetic capacity. Therefore, the metabolic and protein-processing features of *NANS*-high BMDMs provide a plausible transcriptional basis for their enhanced outgoing communication. Functional experiments will be required to determine whether *NANS* directly regulates these programs.

The communication profile of *NANS*-high BMDMs further suggests that this macrophage state may occupy an immunoregulatory and remodeling-oriented niche within the GBM microenvironment. The predominance of LGALS9-centered interactions is particularly relevant because the LGALS9–HAVCR2/TIM-3 axis has been implicated in T-cell dysfunction, macrophage polarization, and immune escape in several tumor contexts, including glioma [17,18,19]. Thus, the enrichment of LGALS9-related outgoing signals from *NANS*-high BMDMs may provide a mechanistic link between this macrophage state and the immunoregulatory phenotype observed in bulk GBM samples. In parallel, PLAU-related signaling may connect *NANS*-high BMDMs to extracellular matrix remodeling, invasion, angiogenesis, and tumor–stromal interaction, processes that are central to GBM progression [20,21]. The concomitant elevation of LGALS9 and PLAU axis scores in *NANS*-high bulk tumors therefore suggests that this state is not simply defined by increased macrophage infiltration, but by a coordinated communication program linking immune suppression with microenvironmental remodeling.

This interpretation is important because immune enrichment in GBM does not necessarily indicate productive anti-tumor immunity. Unlike tumors in which high immune infiltration may reflect an active cytotoxic response, GBM immune infiltration is frequently dominated by myeloid cells, MDSCs, regulatory T cells, and other suppressive components [22,23]. Therefore, the positive association between the *NANS*-high BMDM signature and multiple immune-cell signatures should be interpreted as evidence of an immune-active but potentially suppressive microenvironment. This may also explain why *NANS*-high features are associated with adverse prognosis: increased immune infiltration may reflect macrophage-driven immune remodeling and tumor-supportive inflammation rather than effective tumor clearance.

The drug sensitivity and CMap analyses provide additional translational hypotheses [24]. Compounds predicted to show preferential sensitivity in the *NANS*-high group included inhibitors targeting PI3K/mTOR and MAPK/ERK-related pathways, such as Trametinib, Dasatinib, Ribociclib, PD0325901/Mirdametinib, and Selumetinib. CMap analysis further identified mechanism-of-action classes with negative normalized connectivity scores, including protein synthesis inhibitors, Na+/K+-ATPase inhibitors, exportin antagonists, IGF-1 inhibitors, mTOR inhibitors, PI3K inhibitors, and DNA inhibitors. These findings are conceptually consistent with the metabolic, protein-processing, and stress-response programs observed in *NANS*-high BMDMs. In particular, the convergence of transcriptomic enrichment and drug prediction on mTOR/PI3K-related signaling supports the possibility that this macrophage-associated phenotype is linked to specific metabolic and stress-response dependencies. Beyond direct pathway inhibition, therapeutic reprogramming of tumor-associated macrophages may represent an alternative strategy. Takahashi et al. recently demonstrated that targeting MARCO in combination with anti-CTLA-4 enhanced antitumor efficacy by reprogramming macrophages toward an immune-stimulatory chemokine profile rather than depleting them [25]. These findings raise the possibility that macrophage-directed reprogramming strategies could be combined with PI3K/mTOR- or MAPK/ERK-targeted agents in *NANS*-high GBM, although this hypothesis requires experimental validation.

The radiomics component extends these molecular findings toward noninvasive patient stratification. Tissue-based transcriptomic profiling remains the standard approach for defining immune states, but it is limited by sampling bias, spatial heterogeneity, and the need for surgical tissue. These limitations are particularly relevant in GBM, where immune and stromal features may vary across enhancing tumor, necrotic regions, peritumoral edema, and invasive margins. MRI radiomics provides an opportunity to quantify macroscopic imaging heterogeneity from routinely acquired clinical scans and may therefore serve as a surrogate for selected molecular and immune phenotypes [11,26]. In our study, the association between RadScore and the *NANS*-high BMDM signature suggests that MRI texture and entropy-related features may capture imaging manifestations of macrophage-associated immune and stromal remodeling. However, radiomics should not be interpreted as a direct readout of *NANS* expression or BMDM abundance. Rather, it likely reflects broader tissue-level consequences of this immune state, including enhancement heterogeneity, edema, necrosis, vascular changes, and textural complexity. Therefore, the radiomics model should be viewed as a preliminary imaging biomarker that may support future noninvasive risk stratification after external and prospective validation.

Several limitations should be acknowledged. First, this study was based primarily on public retrospective datasets, including TCGA, CGGA, single-cell reference datasets, and TCIA imaging data. Differences in cohort composition, sequencing platforms, image acquisition protocols, and preprocessing procedures may introduce batch effects and selection bias. Second, although our analyses suggest associations between *NANS*, macrophage metabolic programs, LGALS9/PLAU-centered communication, and immune infiltration, the present study does not provide direct functional validation. Experimental studies are required to determine whether *NANS* regulates sialic acid metabolism, macrophage function, LGALS9/PLAU signaling, or T-cell modulation in GBM. Third, drug sensitivity and connectivity analyses were computational predictions and require validation in relevant preclinical systems. Fourth, although Harmony was applied for batch correction, quantitative batch-mixing evaluation was not performed. Following Harmony-based reclustering and manual annotation, 98.78% of cells retained consistent cell-type annotations compared with the original analysis. Given this high agreement, the original cell annotations and derived signatures were retained for downstream analyses, including CellChat and radiomics, without repeating these analyses. This may potentially affect ligand–receptor inference in CellChat and imaging–transcriptomic associations in the radiomics analysis; therefore, these results should be interpreted with caution. Finally, the radiomics analysis was limited by the small sample size (*n* = 40) relative to the large number of extracted features, resulting in a risk of overfitting. Although elastic net regularization and bootstrap validation were applied, the model should be considered preliminary and exploratory. Independent external validation in larger cohorts is required before clinical application. In addition, tumor volume was not systematically evaluated as a potential correlate of the radiomics score and should be examined in future imaging cohorts.

## 4. Materials and Methods

### 4.1. Study Design and Data Sources

Bulk RNA sequencing data and corresponding clinical information were obtained from the TCGA-GBM cohort and two independent CGGA cohorts (CGGA mRNAseq_693 and CGGA mRNAseq_325) [27]. Single-cell RNA sequencing data from newly diagnosed human glioblastoma samples were obtained from the Brain Immune Atlas (https://www.brainimmuneatlas.org/ (accessed on 1 March 2026)) and GBmap atlas (https://www.omicsdi.org/dataset/geo/GSE211376(accessed on 1 March 2026)) [28]. After merging the datasets, batch correction was performed using Harmony, with dataset identity specified as the batch variable. Harmony-corrected embeddings were used for unsupervised clustering and UMAP visualization. Cell types were subsequently manually annotated based on the expression patterns of canonical marker genes. For radiomics analysis, preoperative MRI data were acquired from the TCIA-GBM collection [29]. From 190 patients with available imaging data, 40 patients with matched transcriptomic data and calculable *NANS*-BMDM signature scores were ultimately included for radiomics model development and validation.

### 4.2. Prognostic Gene Screening and Survival Analysis

Survival-associated genes were identified independently in TCGA-GBM, CGGA mRNAseq_693, and CGGA mRNAseq_325 cohorts. Genes significantly associated with overall survival in each cohort were retained, and overlapping prognostic genes shared by all three datasets were identified. Among these candidates, *NANS* was prioritized for further exploratory investigation based on its macrophage-associated expression pattern, its role in sialic acid biosynthesis, and the limited understanding of its biological significance in GBM. The expression difference of *NANS* between GBM and normal brain tissues was evaluated using GEPIA (http://gepia.cancer-pku.cn/(accessed on 01 March 2026)) [30]. Patients were stratified into high-*NANS* and low-*NANS* groups according to the median *NANS* expression level within each cohort, and overall survival was compared using Kaplan–Meier analysis and the log-rank test.

### 4.3. Single-Cell RNA-Seq Processing and Cell Annotation

Single-cell RNA-seq data were processed using Seurat [14,31]. After quality control, normalization, highly variable gene selection, scaling, principal component analysis, batch correction, clustering, and UMAP visualization, major cell populations were annotated based on canonical marker genes and UMAP localization. Tumor cells were identified using *EGFR*, *SOX2*, *NES*, *BCAN*, *OLIG2*, *PDGFRA*, *CHI3L1*, *CD44*, *VIM*, *MKI67*, and TOP2A; macrophage-lineage cells using *CD68*, *CD163*, *C1QA*, *C1QB*, *C1QC*, *AIF1*, *TYROBP*, *LST1*, *FCER1G*, *CSF1R*, *CD14*, and *FCGR3A*; T cells using *CD3D*, *CD3E*, *TRAC*, *CD2*, *CD4*, *CD8A*, *CD8B*, *NKG7*, and *GZMB*; and oligodendrocyte-lineage cells using *MBP*, *MOG*, *PLP1*, *MAG*, *MOBP*, *CLDN11*, *CNP*, and *SOX10*. Macrophage-lineage cells were further classified into microglia-like tumor-associated macrophages (MG-TAMs) and bone marrow-derived macrophages (BMDMs) [7]. Unsupervised clustering and UMAP visualization in Figure 1D were performed using Harmony-corrected embedding space. Cell populations were subsequently manually annotated based on the expression patterns of canonical marker genes visualized using FeaturePlot. MG-TAMs were identified using *P2RY12*, *TMEM119*, *CX3CR1*, *SALL1*, and *GPR34*, whereas BMDMs were identified using *FCN1*, *S100A8*, *S100A9*, *CCR2*, and *CCL3L3*. Malignant tumor cells were annotated into AC-like, OPC-like, MES-like, and NPC-like states using established GBM cellular-state signatures from Neftel et al., including MES1, MES2, AC, OPC, NPC1, NPC2, G1/S, and G2/M programs [3]. Cell-cycle scores were calculated using ‘CellCycleScoring’, and cycling effects were accounted for during malignant-state classification. T cells were further classified into naïve T, CD4+ T, CD8+ T, and proliferating T-cell subsets based on canonical T-cell lineage and functional markers.

### 4.4. Definition and Characterization of NANS-High BMDMs

BMDMs were extracted for *NANS*-associated analysis. Cells with normalized *NANS* expression = 0 were designated as *NANS*-undetected and were referred to as *NANS*-low for downstream analyses, whereas cells with normalized *NANS* expression > 0 were considered *NANS*-detected. Among *NANS*-detected BMDMs, cells in the upper quartile of *NANS* expression were defined as *NANS*-high BMDMs. Genes upregulated in *NANS*-high BMDMs were used for functional enrichment analysis. Gene Ontology enrichment analysis was performed using clusterProfiler, and Hallmark gene set enrichment analysis was conducted using MSigDB Hallmark gene sets [32,33].

### 4.5. Cell–Cell Communication Analysis

Cell–cell communication analysis was performed using CellChat to compare outgoing signaling programs between *NANS*-high and *NANS*-low BMDMs [34]. CellChat analysis was performed using the original single-cell expression data and corresponding cell-type annotations established before Harmony-based reclustering. *NANS*-high and *NANS*-low BMDMs were analyzed together with annotated malignant tumor states and T-cell subsets. Communication probabilities, pathway-level information flow, and ligand–receptor interactions were inferred using the human CellChat database. Differential communication patterns were compared between *NANS*-high and *NANS*-low BMDMs, with a focus on BMDM-derived signaling toward malignant tumor states and T-cell subsets.

### 4.6. Construction and Validation of the NANS-High BMDM Signature

A *NANS*-high BMDM signature was generated from genes enriched in *NANS*-high BMDMs and applied to TCGA-GBM bulk RNA-seq data. For each TCGA-GBM patient, a *NANS*-BMDM signature score was calculated based on the expression of signature genes. Patients were classified into high-score and low-score groups according to the signature score. Signature gene expression patterns were visualized by heatmap analysis. Immune infiltration scores were estimated using single-sample gene set enrichment analysis (ssGSEA) based on predefined immune cell gene signatures [35]. Spearman correlation analysis was performed to evaluate the association between the *NANS*-BMDM signature score and ssGSEA-derived immune cell scores. Selected CellChat-derived ligand–receptor axes, including LGALS9, PLAU, CCL, and GAS6 signaling axes, were further evaluated in TCGA-GBM bulk transcriptomic data.

### 4.7. Drug Sensitivity and Connectivity Map Analysis

To explore potential therapeutic vulnerabilities associated with the *NANS*-high BMDM phenotype, two drug-related analyses were performed. First, drug response prediction was conducted using the R package ‘oncoPredict’ (version 1.2) to identify compounds with differential predicted sensitivity between *NANS*-high and *NANS*-low tumors [36]. Predicted drug response values were compared between groups using the Wilcoxon rank-sum test, with false discovery rate correction for multiple comparisons, and candidate compounds were visualized using volcano plots according to effect direction and statistical significance. Second, connectivity map analysis was performed using CLUE/CMap normalized connectivity scores to identify compounds and mechanism-of-action classes that may reverse the *NANS*-high macrophage-associated transcriptional program [24]. Negative normalized connectivity scores were interpreted as suggestive of potential transcriptional reversal activity.

### 4.8. MRI Preprocessing and Radiomics Analysis

TCIA MRI data from 40 GBM patients were included for radiomics analysis [37]. IDH mutation status was reviewed for all included patients, among whom 37 were IDH-wildtype and 3 were IDH-mutant. Pre-operative T1 contrast-enhanced and FLAIR images were used for radiomics analysis. All MRI scans were reviewed by three experienced specialists to confirm the tumor regions. Tumor regions of interest were manually delineated using 3D Slicer. T1 contrast-enhanced and FLAIR images were preprocessed and intensity-normalized before radiomics feature extraction and downstream model construction. Image preprocessing included N4 bias field correction, resampling to isotropic 1.0 × 1.0 × 1.0 mm spacing, mask alignment, and per-sequence Z-score intensity normalization [38,39]. Radiomics features were extracted from the delineated tumor regions of interest using PyRadiomics based on the preprocessed MRI images and corresponding tumor masks [40]. Radiomics analysis was performed using TCIA MRI data and matched bulk transcriptomic-derived *NANS*-high BMDM signature scores, with the signature originally established from the single-cell analysis before Harmony-based reclustering. A total of 2553 radiomics features were initially extracted. Features associated with *NANS*-high versus *NANS*-low status were first screened using the Wilcoxon rank-sum test, retaining 309 candidate features. Elastic net logistic regression was then used for feature selection and model construction [41]. Six final radiomics features were selected, and a radiomics score was calculated for each patient. The radiomics score was compared between *NANS*-high and *NANS*-low groups using the Wilcoxon rank-sum test. Spearman correlation analysis was used to evaluate the association between radiomics score and continuous *NANS*-BMDM signature score. Receiver operating characteristic curve analysis was performed to evaluate model performance, and bootstrap validation was used to assess model stability.

### 4.9. Statistical Analysis

Statistical analyses were performed using R (version 4.5.0) and Python (version 2025.2). Group comparisons were conducted using the Wilcoxon rank-sum test. Correlations were assessed using Spearman correlation analysis. Survival differences were evaluated using Kaplan–Meier analysis and the log-rank test. Multiple-testing correction was performed using the Benjamini–Hochberg method. A two-sided *p* value < 0.05 or adjusted *p* value < 0.05 was considered statistically significant.

## 5. Conclusions

In conclusion, this study identifies *NANS*-high BMDMs as a prognostically adverse macrophage-associated state in GBM. This state is characterized by metabolic activation, stress-response and protein-processing programs, intensified communication with tumor and T-cell populations, and LGALS9/PLAU-centered signaling. By linking this macrophage state to bulk immune signatures, predicted therapeutic vulnerabilities, and MRI radiomics, our findings provide a framework for integrating single-cell immunobiology with clinically accessible imaging biomarkers. Future experimental and prospective validation will be essential to determine whether the *NANS*-high BMDM phenotype can guide macrophage-directed or immunometabolic therapeutic strategies in GBM.

## Data Availability

The data supporting the findings of this study are available upon reasonable request from the corresponding author.

## Supplementary Materials

The following supporting information can be downloaded at: https://www.mdpi.com/article/10.3390/ijms27219635/s1.
