# Supplementary material for: Integrative Single-Cell and Radiomics Analyses Identify N-Acetylneuraminate Synthase (NANS)-High Signature Associated with Adverse Prognosis and Immune Remodeling in Glioblastoma
Source: Int J Mol Sci. 2026 Oct 28;27(21):9635. doi: 10.3390/ijms27219635 (PMC13763337; doi:10.3390/ijms27219635)
Supplement: Supplementary file 1 [file ijms-27-09635-s001.zip › ijms-4488352-supplementary.pdf]

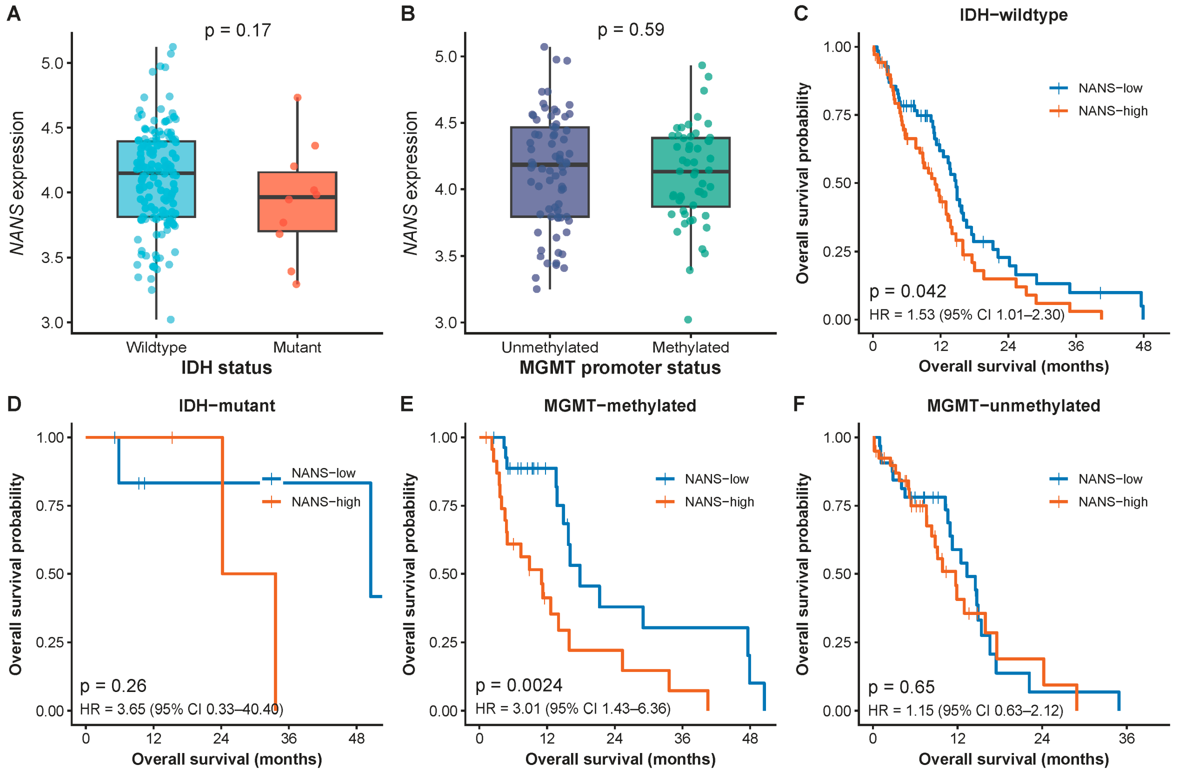

Supplementary Figure S1. Association of *NANS* expression with IDH and MGMT status and stratified survival analysis in TCGA-GBM. (A) Comparison of *NANS* expression between IDH-wildtype and IDH-mutant tumors. (B) Comparison of *NANS* expression between MGMT-unmethylated and MGMT-methylated tumors. (C–F) Kaplan–Meier overall survival analyses according to *NANS* expression in IDH-wildtype, IDH-mutant, MGMT-methylated, and MGMT-unmethylated subgroups, respectively.

Supplementary Table S1. Anonymized summary of prognostic associations and BMDM enrichment status for 41 candidate genes identified across TCGA-GBM and CGGA cohorts.

Gene	Hazard ratio	P value	N	High	Low	BMDM_enriched
NANS	1.504	0.02234	159	79	80	Yes
Gene_01	0.68	0.033	159	79	80	No
Gene_02	1.548	0.01512	159	79	80	No
Gene_03	1.48	0.031	159	79	80	No
Gene_04	1.494	0.02429	159	79	80	No
Gene_05	1.591	0.01171	159	79	80	No
Gene_06	1.84	0.0009253	159	79	80	No
Gene_07	1.447	0.0374	159	79	80	No
Gene_08	1.451	0.0387528	159	79	80	Yes
Gene_09	1.49	0.02481	159	79	80	No
Gene_10	1.455	0.0389	159	79	80	No
Gene_11	1.433	0.0445394	159	79	80	No
Gene_12	1.477	0.02958	159	79	80	Yes
Gene_13	1.503	0.02198	159	79	80	No
Gene_14	1.539	0.01549	159	79	80	Yes
Gene_15	1.435	0.044022	159	79	80	No
Gene_16	1.659	0.00509	159	79	80	Yes

Gene_17	1.709	0.003385	159	79	80	No
Gene_18	1.481	0.02793	159	79	80	No
Gene_19	1.798	0.001185	159	79	80	No
Gene_20	1.526	0.01737	159	79	80	No
Gene_21	1.442	0.040758	159	79	80	No
Gene_22	1.493	0.02587	159	79	80	No
Gene_23	1.471	0.03184	159	79	80	No
Gene_24	1.47	0.03025	159	79	80	No
Gene_25	1.664	0.004213	159	79	80	No
Gene_26	1.426	0.0452	159	79	80	No
Gene_27	1.683	0.00371	159	79	80	Yes
Gene_28	0.607	0.005301	159	79	80	No
Gene_29	1.537	0.01615	159	79	80	Yes
Gene_30	1.524	0.01902	159	79	80	No
Gene_31	1.494	0.02384	159	79	80	No
Gene_32	1.792	0.001026	159	79	80	No
Gene_33	0.697	0.04466	159	79	80	No
Gene_34	1.558	0.01237	159	79	80	Yes
Gene_35	1.412	0.049833	159	79	80	No
Gene_36	0.653	0.01692	159	79	80	Yes
Gene_37	1.525	0.02158	159	79	80	No
Gene_38	1.596	0.008463	159	79	80	No
Gene_39	1.558	0.01472	159	79	80	Yes
Gene_40	1.614	0.008277	159	79	80	Yes
